# Factors associated with the status of usual source of care during the COVID-19 pandemic: a nationwide survey in Japan

**DOI:** 10.1186/s12875-023-02148-9

**Published:** 2023-09-27

**Authors:** Takuya Aoki, Masato Matsushima

**Affiliations:** 1 Division of Clinical Epidemiology, Research Center for Medical Sciences, The Jikei University School of Medicine, 3-28-5, Nishishimbashi, Minato-ku, Tokyo, 105-8461 Japan; 2 Section of Clinical Epidemiology, Department of Community Medicine, Graduate School of Medicine, Kyoto University, 54 Kawahara-cho, Syogoin, Sakyo-ku, Kyoto, 606-8507 Japan

**Keywords:** covid-19, Delivery of health care, Health literacy, Japan, Primary health care, Social isolation

## Abstract

**Background:**

To ensure that high-quality primary care is available to every individual, increasing the proportion of residents with a usual source of care (USC) is a challenge for each country. However, the status of USC after the spread of COVID-19 and the factors associated with it remain unclear internationally. Therefore, we aimed to explore the associations of sociodemographic and clinical factors with the presence and type of USC (*kakaritsukei* in Japanese) during the pandemic in Japan.

**Methods:**

We conducted a nationwide cross-sectional survey of a representative sample of the general Japanese adult population in May 2021. The main outcome measures were the presence and type of USC. We assessed sociodemographic and clinical factors, including age, gender, marital status, years of education, employment status, annual household income, social isolation, health literacy, number of chronic conditions, and health-related quality of life.

**Results:**

Of the 1,757 participants, 1,011 (57.5%) had a USC. There were 769 (76.1%) participants who had a USC in a clinic and 227 (22.5%) in a hospital. As a result of multivariable modified Poisson regression analysis, male gender, no chronic condition, lower health literacy, and social isolation were significantly associated with not having a USC. Among participants with a USC, male gender, the presence of one or more chronic conditions, and lower health-related quality of life were associated with having a hospital-based USC.

**Conclusions:**

We identified factors associated with the status of USC during the COVID-19 pandemic, including health literacy and social isolation. These findings provide primary care providers and policymakers with insight into the potential barriers to having a USC in the aftermath of the pandemic.

## Background

A usual source of care (USC) is a person or place that people can turn to with a health issue or concern and is generally considered primary care [1]. There is a wealth of evidence regarding the benefits of having a USC. For example, having a USC has been associated with improved receipt of preventive services, [2] better control of chronic diseases, [3] more patient-centered communication, [4] and lower health care costs [5]. Furthermore, even during the coronavirus disease 2019 (COVID-19) pandemic when there were many barriers to providing usual medical care, the presence of USC was associated with increased preventive care utilization and decreased total hospitalization [6, 7].

To ensure that high-quality primary care is available to every individual, increasing the proportion of residents with a USC is a challenge for each country. In the United States, the Primary Care Collaborative Evidence Report showed that the percentage of Americans with a USC has been declining, falling 10% between 2000 and 2019, from 84 to 74%. In 2020, there was a slight uptick in USC to 75%.^1^ In Japan, the proportion of adult residents with USC, called *kakaritsukei* in Japanese, was about 55% in 2017 and 2020, [8] which is lower than in the United States, even though the Ministry of Health, Labour and Welfare has recommended that everyone should have a USC.

In Japan, the pandemic has highlighted issues related to primary care, including ambiguity about the role of USC. For example, a previous study found that approximately one-fifth of Japanese adult residents who had a USC reported limited access to primary care for COVID-19 consultation due to their USC’s refusal during the pandemic [9]. The Japanese government recognized the need to clarify and strengthen the role of primary care physicians and decided to legislate the function of the *kakaritsukei* in 2022 [10]. Further dissemination of USC and its contribution is required in Japan, but in order to do so, it is important to clarify the factors associated with the status of USC after the COVID-19 expansion.

In studies in other countries such as the United States and Korea before the COVID-19 pandemic, several sociodemographic and clinical factors have been identified as being associated with the presence of USC [11–13]. These include age, gender, marital status, region, education, race, income, insurance, and chronic conditions. Similarly, a survey conducted by the Japan Medical Association Research Institute reported that older adults and women were more likely to have a USC [8]. However, the status of USC after the spread of COVID-19 and the factors associated with it remain unclear internationally. Therefore, in the present study, we aimed to explore the associations of sociodemographic and clinical factors with the presence and type of USC during the pandemic in Japan. We were particularly interested in the factors of health literacy and social isolation, which are major health issues worldwide as a result of the pandemic [14, 15]. Clarifying them will be useful for improving primary care system after the pandemic.

## Methods

### Design, setting, and participants

We used the data from the baseline survey of the National Usual source of Care Survey (NUCS). The NUCS was a nationwide mail survey collecting USC, health care utilization, health status, and sociodemographic data from a representative sample of Japanese adults. The baseline survey was conducted in May 2021. Details of the NUCS, including sampling methods, have been reported previously [16]. In summary, 2,000 potential participants between the ages of 20 and 75 were selected from a nationally representative panel using stratified random sampling. The sample size was consistent with the needs of other studies that are part of the NUCS. Participants in the survey were given JPY 500 gift certificates.

### Measures

#### Usual source of care

In Japan, physicians who have completed their training in internal medicine and then work in clinics or small and medium-sized hospitals with fewer than 200 beds often serve as USCs [8, 17]. In addition to them, the Japan Primary Care Association has been certifying family physicians since 2010, and the Japanese Medical Specialty Board launched a new certification program for primary care specialists in 2018, but the number of these specialists is still small.

To determine an individual’s USC, the following item was used in the Japanese version of Primary Care Assessment Tool [18, 19] and nationwide surveys, such as the Medical Expenditure Panel Survey: “Is there a doctor that you usually go to if you are sick or need advice about your health?”. Participants’ responses were on a binary scale (yes vs. no). If participants answered yes to the primary question, they were asked to answer the following item on the type of their USC: “Where does the doctor work?” Participants were asked to answer clinic, hospital, university hospital, or other. A participant was considered to have a USC if he or she was able to identify a physician practicing outside of university hospitals. This is because the Ministry of Health, Labour and Welfare of Japan separates the roles of *kakaritsukei*, which provide primary care, and university hospitals.

### Sociodemographic and clinical factors

We collected data on the sociodemographic and clinical factors of the participants. The structured questionnaire measured the age, gender, marital status, years of education, employment status, annual household income, social isolation assessed by the Japanese version of the abbreviated Lubben Social Network Scale (LSNS-6), [20] health literacy assessed by the Communicative and Critical Health Literacy (CCHL), [21] number of chronic conditions, and health-related quality of life assessed by the five-level version of the EuroQol five-dimensional questionnaire (EQ-5D-5 L) [22].

The LSNS-6 is a 6-item self-reported scale used to assess social isolation by measuring perceived social support received from family and friends. This tool assesses the size, closeness, and frequency of contacts in a respondent’s social network. The scores range from 0 to 30 points, with higher scores indicating a better quality of social network. We classified participants with an LSNS-6 score < 12 points as socially isolated, as suggested in the previous study [23].

The CCHL consists of five items and the scores range from 1 to 5 points, with higher scores indicating higher health literacy. The CCHL items were constructed to directly reflect the World Health Organization’s definitions of communicative and critical health literacy [21].

The EQ-5D-5 L is a generic instrument for describing and valuing health. It is based on a descriptive system that defines health in terms of 5 dimensions: Mobility, Self- Care, Usual Activities, Pain/Discomfort, and Anxiety/Depression. Higher scores indicate better health-related quality of life [22].

We used a validated list of 20 chronic conditions that was developed based on previous literature on multimorbidity and its relevance to the primary care population [24]: hypertension, depression/anxiety, chronic musculoskeletal conditions that cause pain or limitation, arthritis/rheumatoid arthritis, osteoporosis, chronic respiratory disease (asthma, chronic obstructive pulmonary disease, or chronic bronchitis), cardiovascular disease, heart failure, stroke/transient ischemic attack, stomach problems, colon problems, chronic hepatitis, diabetes, thyroid disorder, any cancer in the past 5 years, kidney disease/failure, chronic urinary problem, dementia/Alzheimer’s disease, hyperlipidemia, and obesity.

### Statistical analysis

We calculated the descriptive statistics for individual sociodemographic and clinical factors by USC status. Multivariable modified Poisson regression analysis with robust error variance was conducted to determine the associations of sociodemographic and clinical factors with the presence of USC during the pandemic. We also performed an additional modified Poisson regression analysis to explore the associations of sociodemographic and clinical factors with the type of USC (clinic-based or hospital-based) among participants who had a USC. In the analysis, we excluded the small number of participants whose USC worked in settings other than clinics and hospitals.

For each analysis, we used a two-sided significance level of *P* = 0.05. We performed complete case analyses for missing independent variables in the regression models because the missing data were low (0–4.0%). Statistical analyses were conducted using R, version 4.2.1 (R Foundation for Statistical Computing, Vienna, Austria; www.R-project.org).

## Results

### Participant characteristics

Of the 2,000 adult residents, 1,757 completed the NUCS baseline survey (response rate: 87.9%). Of the participants, 1,011 (57.5%) had a USC. There were 769 (76.1%) participants who had a USC in a clinic, 227 (22.5%) in a hospital, and 15 (1.5%) in other settings or were missing. Table 1 shows the characteristics of the study population by USC status. Compared with participants without a USC, those with a USC were older (≥ 70 years, 15.4% vs. 5.1%), more often female (53.9% vs. 47.3%), more often unemployed (29.7% vs. 20.2%), less often socially isolated (26.6% vs. 35.7%), and had more chronic conditions (with ≥ 2 chronic conditions, 34.5% vs. 11.9%).

**Table 1:** Participants’ characteristics with or without usual source of care

	Total	Has USC	No USC
Characteristic	(N = 1,757)	(n = 1,011)	(n = 746)
Age, n (%), years			
20 − 29	202 (11.5)	95 (9.4)	107 (14.3)
30 − 39	293 (16.7)	136 (13.5)	157 (21.0)
40 − 49	364 (20.7)	164 (16.2)	200 (26.8)
50 − 59	325 (18.5)	198 (19.6)	127 (17.0)
60 − 69	379 (21.6)	262 (25.9)	117 (15.7)
≥ 70	194 (11.0)	156 (15.4)	38 (5.1)
Gender, n (%)			
Female	898 (51.1)	545 (53.9)	353 (47.3)
Marital status, n (%)			
Married	1,333 (75.9)	784 (77.5)	549 (73.6)
Widowed	65 (3.7)	45 (4.5)	20 (2.7)
Annulled, divorced, separated	89 (5.1)	55 (5.4)	34 (4.6)
Never married	269 (15.3)	127 (12.6)	142 (19.0)
Data missing	1 (0.1)	0 (0.0)	1 (0.1)
Education, n (%)			
Less than high school	57 (3.2)	37 (3.7)	20 (2.7)
High school	584 (33.2)	348 (34.4)	236 (31.6)
Junior college	484 (27.5)	279 (27.6)	205 (27.5)
More than or equal to college	590 (33.6)	323 (31.9)	267 (35.8)
Data missing	42 (2.4)	24 (2.4)	18 (2.4)
Employment status, n (%)			
Full-time employee	691 (39.3)	347 (34.3)	344 (46.1)
Part-time employee	387 (22.0)	231 (22.8)	156 (20.9)
Self employee	227 (12.9)	132 (13.1)	95 (12.7)
Unemployed	451 (25.7)	300 (29.7)	151 (20.2)
Data missing	1 (0.1)	1 (0.1)	0 (0.0)
Annual household income, n (%), million JPY			
< 3.00 (≒ 22,000 US dollars)	288 (16.4)	170 (16.8)	118 (15.8)
3.00–4.99	532 (30.3)	332 (32.8)	200 (26.8)
5.00–6.99	435 (24.8)	256 (25.3)	179 (24.0)
7.00–9.99	312 (17.8)	167 (16.5)	145 (19.4)
≥ 10.00	170 (9.7)	76 (7.5)	94 (12.6)
Data missing	20 (1.1)	10 (1.0)	10 (1.3)
Social isolation, n (%)			
Absent	1210 (68.9)	736 (72.8)	474 (63.5)
Present	535 (30.4)	269 (26.6)	266 (35.7)
Data missing	12 (0.7)	6 (0.6)	6 (0.8)
CCHL, mean (SD)	3.5 (0.7)	3.5 (0.7)	3.5 (0.7)
Data missing, n (%)	8 (0.5)	3 (0.3)	5 (0.7)
Number of chronic conditions, n (%)			
0	794 (45.2)	324 (32.0)	470 (63.0)
1	454 (25.8)	297 (29.4)	157 (21.0)
≥ 2	438 (24.9)	349 (34.5)	89 (11.9)
Data missing	71 (4.0)	41 (4.1)	30 (4.0)
EQ-5D-5 L, mean (SD)	0.89 (0.08)	0.88 (0.09)	0.90 (0.07)
Data missing, n (%)	7 (0.4)	2 (0.2)	5 (0.7)

Abbreviations: USC, usual source of care, CCHL, Communicative and Critical Health Literacy; EQ-5D-5 L, five-level version of the EuroQol five-dimensional questionnaire

## Factors associated with the presence of USC

**Fig. 1:**
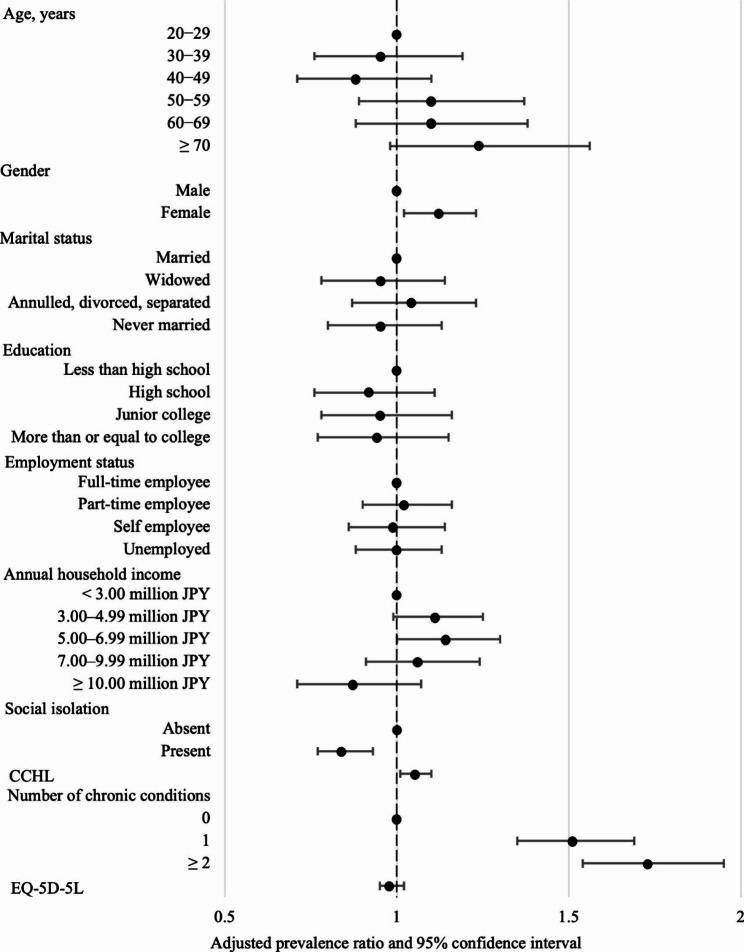
Factors associated with the presence of a usual source of care (n = 1,621). Abbreviations: CCHL, Communicative and Critical Health Literacy; EQ-5D-5 L, five-level version of the EuroQol five-dimensional questionnaire. CCHL and EQ-5D-5 L: prevalence ratio per 1SD increase in score

Figure 1 shows the result of multivariable modified Poisson regression analysis, exploring the associations of sociodemographic and clinical factors with the presence of USC during the COVID-19 pandemic. A total of 1,621 subjects were included in the multivariable analysis. Gender, social isolation, CCHL score, and the number of chronic conditions were significantly associated with the presence of USC. Adjusted prevalence ratios of having a USC were 1.12 (95% confidence interval (CI): 1.02–1.23) for being female (vs. male), 0.84 (95%CI: 0.77–0.93) for being socially isolated (vs. not socially isolated), 1.05 (95%CI: 1.01–1.10) per 1SD increase in CCHL score, and 1.51 (95%CI: 1.35–1.69) for the presence (vs. absence) of a chronic condition.

### Factors associated with the type of USC

Table 2 shows the result of multivariable modified Poisson regression analysis, exploring the associations of sociodemographic and clinical factors with the type of USC (clinic-based or hospital-based). A total of 916 subjects with USC and no missing independent variables were included in the multivariable analysis. As a result, male gender, the presence of one or more chronic conditions, and lower EQ-5D-5 L scores were associated with having a hospital-based USC.

**Table 2:** Factors associated with having a hospital-based usual source of care (n = 916)

	aPR (95%CI)	*P* value
Age, years		
20 − 29	ref	
30 − 39	0.56 (0.28 − 1.12)	0.100
40 − 49	0.45 (0.23 − 0.90)^b^	0.024
50 − 59	0.77 (0.41 − 1.43)	0.400
60 − 69	0.91 (0.47 − 1.78)	0.791
≥ 70	0.94 (0.47 − 1.91)	0.872
Gender		
Male	ref	
Female	0.71 (0.54 − 0.94)^b^	0.018
Marital status		
Married	ref	
Widowed	0.88 (0.50 − 1.57)	0.673
Annulled, divorced, separated	0.94 (0.56 − 1.56)	0.802
Never married	0.95 (0.57 − 1.57)	0.828
Education		
Less than high school	ref	
High school	0.82 (0.51 − 1.32)	0.419
Junior college	1.03 (0.62 − 1.71)	0.906
More than or equal to college	0.64 (0.39 − 1.06)	0.081
Employment status		
Full-time employee	ref	
Part-time employee	0.82 (0.55 − 1.21)	0.317
Self employee	1.01 (0.69 − 1.47)	0.966
Unemployed	0.87 (0.60 − 1.26)	0.466
Annual household income, million JPY		
< 3.00 (≒ 22,000 US dollars)	ref	
3.00–4.99	0.99 (0.70 − 1.41)	0.957
5.00–6.99	1.04 (0.70 − 1.53)	0.845
7.00–9.99	0.74 (0.44 − 1.22)	0.236
≥ 10.00	0.98 (0.54 − 1.76)	0.935
Social isolation		
Absent	ref	
Present	1.14 (0.89 − 1.47)	0.306
CCHL^a^	0.99 (0.87 − 1.12)	0.802
Number of chronic conditions		
0	ref	
1	1.73 (1.20 − 2.49)^b^	0.003
≥ 2	1.64 (1.13 − 2.38)^b^	0.010
EQ-5D-5L^a^	0.89 (0.83 − 0.97)^b^	0.006

Abbreviations: aPR, adjusted prevalence ratio; CCHL, Communicative and Critical Health Literacy; EQ-5D-5 L, five-level version of the EuroQol five-dimensional questionnaire

^a^Prevalence ratio per 1SD increase in score

^b^Significant difference, P < 0.05

## Discussion

The nationwide study of the Japanese adult population revealed that sociodemographic and clinical factors were differentially associated with the status of USC even during the COVID-19 pandemic. To increase Japanese adults who have a USC, an approach that targets residents who are male, without chronic conditions, with lower health literacy, and with social isolation may be warranted. In addition, our study also found that factors such as gender, the presence of chronic conditions, and health-related quality of life were associated with the type of USC. This study provided additional insight into USC because of changes in the social environment and health care utilization internationally after the COVID-19 expansion.

The proportion of adult residents with USC in this study was similar to that in a recent survey conducted by the Japan Medical Association Research Institute and did not increase after the expansion of COVID-19 [25]. Despite the increased importance of primary care due to the COVID-19 pandemic, more than 40% of adults still did not have a USC. Japan’s long-standing system of free access to specialists may be one of the barriers to the continuity of care and the spread of USC. Japanese policymakers need to take fundamental steps to strengthen the primary care system after the pandemic.

Our findings were consistent with previous studies conducted before the COVID-19 pandemic, which showed that adults with a USC were more likely to be female and to have chronic conditions [11–13]. Our results showed that older residents were more likely to have a USC, but the difference was not statistically significant after adjusting for other factors. Prior studies in the United States have reported that residents with longer educational years and higher income were also more likely to have a USC, but no similar association was found in Japan, which has a universal health insurance system that provides comprehensive coverage to all Japanese citizens [26].

Although the association between health literacy and the presence of USC had been poorly studied and unclear, [27] in our study, the association was statistically significant in the multivariable analysis. Lower health literacy may be a barrier to having USC, as well as other positive health behaviors. In addition, the present study also reported the association between social isolation, which is a major health problem and has increased worldwide due to the pandemic, and the presence of USC. The primary care utilization of socially isolated people remains unclear worldwide. A previous study conducted in Canada before the pandemic suggested an association between social capital and having a regular family doctor; however, the study used a measure of social capital that has not been examined for reliability and validity [28]. Our findings regarding USC provide additional information about potential mechanisms by which social isolation influences health outcomes. Although the Japanese government plans to disseminate USC by providing residents with information on the functions of each *kakaritsukei* through its website and other sources, there will be a need to reach out to vulnerable populations who are socially isolated or have low levels of health literacy and need primary care supports.

Our study also showed that adults with a chronic condition or poorer health status are more likely to have a hospital-based USC. These results can be attributed to Japan’s free access system and the fact that hospitals have more medical equipment than clinics and provide inpatient care in addition to outpatient care. The previous study in the United States did not report similar results regarding differences in patient characteristics between clinic-based and hospital-based USC [29]. Our results may be useful in considering the shared roles and coordination between clinics and small and medium-sized hospitals in Japan, where primary care is provided in a variety of settings.

A key strength of our study is the use of data from a nationwide study, with a sample representative of the Japanese adult population, which allows for the generalization of its results to the wider population. Another strength of this study is the high response rate. However, the present study has several potential limitations. First, in this study, we defined a USC as a specific doctor and collected the information by using a structured questionnaire. In other countries, a USC may be considered a specific person or a specific place, but in Japan, it currently refers to a specific doctor according to the Ministry of Health, Labour and Welfare. Therefore, caution should be exercised when comparing our findings with those of other countries. Second, we used a validated list of chronic conditions; however, self-reported data for identifying chronic conditions may have introduced misclassification bias.　Third, it is difficult to compare the results of the present study with pre-pandemic results because no previous nationwide study that comprehensively investigated the factors associated with the status of USC exists in Japan. Fourth, given the cross-sectional nature of the data, causal relationships of the identified factors with the presence and type of USC cannot be definitively established. Fifth, although we used a national panel based on a multistage sampling method, caution should be exercised in generalizing the results of this study due to the nature of panel surveys.

## Conclusions

We identified factors associated with the status of USC during the COVID-19 pandemic, including health literacy and social isolation. These findings provide primary care providers and policymakers with insight into the potential barriers to having a USC in the aftermath of the pandemic.

## Data Availability

The datasets generated and analyzed in the current study are not publicly available because we did not obtain informed consent from the participants to share the data. However, the datasets are available from the corresponding author upon reasonable request.

## Abbreviations

USC: Usual source of care
COVID-19: Coronavirus disease 2019
NUCS: National Usual source of Care Survey
LSNS-6: Lubben Social Network Scale
CCHL: Communicative and Critical Health Literacy
EQ-5D-5 L: Five-level version of the EuroQol five-dimensional questionnaire
